# New Appliances and Things Medical

**Published:** 1894-12-01

**Authors:** 


### NEW APPLIANCES AND THINGS MEDICAL.

### THE VIKING NORWEGIAN CONDENSED MILK.

(The Norwegian Milk Condensing Company. Agents: Johnsen and Jorgensen, Botolph House, Eastcheap, London, E.C.)

Hitherto the condensed milks introduced into this country have nearly all contained added sugar, or other preservative, and therefore have not represented the natural cow's milk when diluted with water. In many cases also these condensed milks have been made from skimmed milk, and have consequently been deficient in cream. Even at the present time there are many such condensed skimmed milks in the market, and the label announcing this fact is frequently printed in such small type that purchasers are often misled into buying these comparatively worthless articles. We have submitted the samples of "Viking" condensed milk to analysis, and find that the above statements do not apply to it, as we have found that no cane sugar nor other preservative has been added, and that it contains the whole of the fat present in a genuine milk. This result has been obtained by condensing the fresh milk without the addition of any foreign substance by an entirely new process devised by Dr. Olaf J. Olsen to remedy the defects which obtain in the older forms of condensing plant. In appearance, flavour, and keeping properties it closely resembles fresh milk when thinned, and its absolute freedom from the common milk fungus as well as from bacteria and pathogenic organisms is guaranteed by the agents. We are also pleased to note that the proprietors have represented accurately the amount of water which it is necessary to add in order to thin it to the consistency and strength of normal milk, so that whenever this preparation is used there is no likelihood of children being starved from following the absurd instructions which are sometimes met with on condensed milk tins. It should prove of considerable value for children and invalids when good fresh milk is unattainable and is to be preferred to that commonly met with in our large towns.

### IMPROVED ARCH SUPPORTS FOR THE FEET.

(James Pond, 23, Castle Meadow, Norwich.)

Various forms of valgus pad and arch support have appeared from time to time. Some have answered their purpose, others have been too uncomfortable to wear continuously, and others have soon got out of place or otherwise misshapen. The arch support which we have before us appears to possess many excellent qualities. It takes the form of an artificial sole, with a valgus pad of good steel to support the inferior surfaces of the cuneiform and navicular bones. This is a point of special importance, as other varieties of support occasionally only impinge on the inner side of the foot, and are ineffectual for the purpose intended. Pond's arch supports are as good a thing of the kind as we have ever met with.

